# Pill Burden: A Quality-of-Life Measure After Parathyroidectomy for Secondary Hyperparathyroidism

**DOI:** 10.1097/AS9.0000000000000644

**Published:** 2026-01-23

**Authors:** Rachael C. Acker, Jasmine Hwang, Shane Williams, Heather Wachtel, Douglas Fraker, Gary E. Weissman, Yuvaram Reddy, Rachel R. Kelz

**Affiliations:** From the * Department of Surgery, University of Pennsylvania Health System, Philadelphia, PA; † Renal-Electrolyte and Hypertension Division, Department of Medicine, Leonard Davis Institute of Health Economics, University of Pennsylvania, Philadelphia, PA; ‡ Renal-Electrolyte and Hypertension Division, Department of Medicine, Center for Surgery and Health Economics, University of Pennsylvania, Philadelphia, PA; § Department of Medicine, University of Pennsylvania Health System, Philadelphia, PA; ‖ Renal-Electrolyte and Hypertension Division, Department of Medicine, Hospital of the University of Pennsylvania, Philadelphia, PA; ¶ Corporal Michael J. Crescenz VA Medical Center, Philadelphia, PA.

## INTRODUCTION

Secondary hyperparathyroidism (SHPT), a condition that affects most dialysis patients, is associated with vascular calcifications and increased risk of cardiovascular mortality. While most cases are managed medically, many patients are referred to surgery because of high parathyroid hormone (PTH) levels refractory to medication or due to medication side effects or nonadherence. Both medical and surgical management are associated with high pill burdens that negatively impact quality of life in dialysis patients.^1^ To improve preoperative counseling and shared decision-making, the aim of this study was to compare the pre and postoperative daily pill burdens of patients on dialysis undergoing parathyroidectomy and to examine the relationship between preoperative PTH levels and postoperative pill burden.

## METHODS

This retrospective cohort study used electronic health record data of adult patients with kidney failure on dialysis admitted after index parathyroidectomy (July 2017–April 2025) at a single, high-volume academic hospital. Patients were identified using the Complete Inpatient Record Using Comprehensive Electronic database—a platform within our health system designed to capture and share clinically validated healthcare data.^2^

A systematic approach to manual chart review was established (R.C.A., J.H., and R.R.K.) and performed by 1 author (R.C.A.) after agreement on the data definitions. Dialysis modality, age, sex, ethnicity, race, insurance status/payer, indication for parathyroidectomy, Elixhauser comorbidities,^3^ and lab values were extracted.

Because the 2022 American Association of Endocrine Surgeons guideline lacks a biochemical threshold for parathyroidectomy,^4^ the primary exposure in this study was defined by the median preoperative PTH level (140.6 pmol/L or 1325.9 pg/mL). Day of surgery levels were used to compare patients off of calcimimetic treatment. Patients were stratified into 2 groups—high or low.

The outcome was postoperative treatment-related daily pill burden—the number of pills prescribed per day for calcium homeostasis. The primary focus was pill burden at discharge. Secondarily, pill burden at 6 months was examined. Total pill burden included medications for other conditions.

We used Welch *T* Test, χ^2^, Generalized Wilcoxon, and Fisher Exact tests for univariate analyses, R 4.41 (R Core Team, Austria, Vienna). This study was exempted by the University of Pennsylvania Institutional Review Board (protocol #857117).

## RESULTS

Patients in the low and high PTH level groups (n = 64) had similar characteristics (Table 1). Preoperatively, there were no differences in the daily pill burdens between low and high PTH groups (Table 2). The median postoperative PTH level was 19.9 pmol/L (Interquartile range: 1.6, 74.8) (equivalent to 187.7 pg/mL). The median length of stay was 3.8 days (2.9, 5.5) without differences between groups (3.3 vs 4.2 days, *P* = 0.08). At discharge, the median treatment-related daily pill burden was 27 pills (23, 36; range: 4–69 pills) with a significantly lower burden in the low compared with high PTH group (24 vs 32 pills, *P* = 0.01). At 6 months (n = 33), the treatment-related daily pill burden was similar between groups (Table 2). Compared with preoperative regimens, daily pill burdens increased by a median of 23 (18, 31) pills at discharge and by 5 pills (−1, 11) 6 months after surgery.

**TABLE 1.** Patient Demographics and Laboratory Values by Low and High Preoperative Parathyroid Hormone (PTH) Levels Among Patients With Secondary Hyperparathyroidism (SHPT)

Characteristic No. (%)	All Patients n = 64	Low Preoperative PTH, <140.6 pmol/L n = 32	High Preoperative PTH, ≥140.6 pmol/L n = 32	*P*
Age median (IQI)	52 (42, 59)	53 (46, 60)	49 (39, 55)	0.20
Dialysis type				
Peritoneal dialysis	10 (16)	6 (19)	4 (13)	0.73
Hemodialysis	54 (84)	26 (81)	28 (88)	
Operation (type of parathyroidectomy: subtotal or total with autotransplantation)				
Subtotal, yes	63 (98)	32 (100)	31 (97)	1.00
Sum of comorbidities median (IQI)	5 (4, 7)	6 (4, 8)	5 (4, 5)	0.08
Etiology of renal disease				
Hypertension	27 (42)	13 (41)	14 (44)	1.00
Diabetic nephropathy	13 (20)	9 (28)	4 (13)	0.21
Focal segmental glomerulosclerosis	8 (13)	3 (9)	5 (16)	0.71
Polycystic kidney disease	3 (5)	1 (3)	2 (6)	1.00
Glomerulonephritis	3 (5)	3 (9)	0	0.24
Other	17 (27)	8 (25)	9 (28)	1.00
Years on dialysis	6 (4, 7)	5 (3, 7)	6 (5, 8)	0.08
Indication for surgery				
Biochemical	40 (63)	17 (53)	23 (72)	0.20
Medication-related	14 (22)	9 (28)	5 (16)	0.36
Calciphylaxis	4 (6)	3 (9)	1 (3)	0.61
Other SHPT symptoms	17 (27)	7 (22)	10 (31)	0.57
Preoperative labs (mg/dL) median (min, max)				
Calcium	9.0 (6.4, 10.8)	9.0 (6.4, 10.5)	9.0 (7.6, 10.8)	0.52
Phosphate	6.5 (2.7, 10.8)	6.7 (2.7, 10.8)	6.1 (2.8, 10.1)	0.58
Alkaline phosphatase	199 (30, 1953)	140 (30, 579)	286 (91, 1953)	**0.001**
Intraoperative labs (pmol/L) median (min, max)				
Starting PTH value	140.6 (23.9, 349.9)	106.6 (23.9, 140.3)	174.7 (140.8, 349.9)	**<0.001**
Ending PTH	19.9 (1.6, 74.8)	17.1 (1.6, 45.2)	22.7 (6.0, 74.8)	**0.01**
Change in PTH (Absolute value)	113.7 (18.7, 334.3)	85.0 (18.7, 117.5)	157.3 (117.4, 334.3)	**<0.001**
Postoperative labs (mg/dL) Median (min, max)				
Calcium 0–24 hours	8.0 (6.5, 9.9)	8.1 (6.5, 9.9)	7.8 (6.7, 9.5)	0.14
Calcium 24–48 hours	8.3 (6.7, 11.0)	8.6 (7.0, 11.0)	8.1 (6.7, 9.8)	**0.003**
Calcium 48–72 hours	8.4 (6.7, 10.7)	8.5 (7.4, 10.6)	7.6 (6.7, 10.7)	0.08
Calcium ≥72 hours	8.3 (6.8, 11.3)	8.7 (7.2, 11.3)	8.0 (6.8, 11.1)	0.20

There were no significant differences in the proportion of patients by sex, race, BMI, ethnicity, or insurance status between groups. Out of 31 comorbidities examined, the only significant difference between groups was diabetes with a complication (low PTH: 47% vs high PTH: 17%, *P* = 0.02). There were no differences in postoperative phosphate or alkaline phosphatase between groups. IQI, Interquartile Interval.

Bolded values represent *P*-values <0.05.

**TABLE 2.** Selected Pre and Postoperative Medication and Doses

Median [IQI] (min, max)	All Patients n = 64	Low Preoperative PTH n = 32	High Preoperative PTH n = 32	*P*
**Preoperative**				
**Medications**				
Cinacalcet (mg)	90 [60, 90] (30, 180)	90 [60, 90] (30, 180)	90 [60, 120] (30, 180)	0.98
Sevelamer (mg)	2400 [2400, 2400] (800, 7200)	2400 [2400, 2400] (800, 4800)	2400 [2400, 2400] (1600, 7200)	1.00
Calcium acetate (mg)	2001 [1334, 4002] (60, 6003)	1334 [667, 4002] (60, 6003)	2335 [2001, 4002] (667, 6003)	0.35
**Pill Burden (number of pills per day**)				
Treatment-related	4 [2, 7] (6, 11)	3 [2, 6.5] (6, 11)	5 [3, 7] (8, 11)	0.16
Other	8 [6, 11] (2, 20)	10 [7, 11] (2, 20)	8 [6, 10] (2, 18)	0.34
Total	13 [10, 18] (0, 14)	14 [11, 18] (0, 14)	12 [10, 18] (0, 13)	0.75
Postoperative				
**Medications at discharge**				
Calcitriol (µg)	4.5 [4, 6] (0.5, 14.0)	4 [3.8, 5.1] (0.5, 14.0)	5.0 [4.0, 7.0] (1.0, 11.0)	**0.01**
Calcium Carbonate (g)	7.5 [4.5, 9.0] (1.5, 20.0)	6.0 [4.1, 8.0] (1.5, 14.0)	8.0 [6.0, 12.0] (3.0, 20.0)	**0.05**
Sevelamer (mg)	2400 [2400, 4800] (2400, 12000)	2400 [2400, 6000] (2400, 12000)	2400 [2400, 3600] (2400, 7200)	0.75
Calcium acetate (mg)	4002 [2001, 4002] (667, 6003)	2001 [2001, 2001] (2001, 4002)	4002 [4002, 4002] (667, 6003)	**0.03**
**Pill Burden at discharge (number of pills per day**)				
Treatment-related	27 [23, 36] (4, 69)	24 [16, 33] (4, 49)	32 [26, 37] (12, 69)	**0.01**
Other	8 [6, 11] (2, 20)	10 [7, 11] (2, 20)	8 [6, 10] (2, 18)	0.34
Total	35 [30, 44] (10, 85)	32 [27, 41] (10, 60)	42 [34, 45] (20, 85)	**0.04**
**Pill Burden at 6 months (number of pills per day**)				
Number of patients with 6 month follow up data	n = 33	n = 18	n = 15	
Treatment-related	10 [4, 21]	9 [3, 23]	10 [5, 16]	0.71
Other	10 [7, 12]	10 [7, 13]	9 [7, 11]	0.65
Total	20 [13, 28]	19.5 [14, 27]	20 [14, 28]	0.71

There were no differences in the proportion of patients prescribed each type of medication between groups. Postoperative medications were captured at the time of discharge. *P* values compare median daily pill burdens between low and high PTH groups using Generalized Wilcoxon tests. A total of 33 patients (52%) had encounters in our health system 6 months after surgery with medication reconciliations. IQI, Interquartile interval; Min, minimum; Max, maximum. Bolded values indicate statistically significant *P* values.

## DISCUSSION

After parathyroidectomy for SHPT, median treatment-related daily pill burden increases by 23 pills at discharge and 5 pills by 6 months. At discharge, patients with lower compared with higher preoperative PTH have smaller pill burden increases. This difference resolves by 6 months.

Similar to published studies, the preoperative PTH levels in our cohort were significantly higher than the historical biochemical threshold for parathyroidectomy (PTH ≥84.8 pmol/L, equivalent to 800 pg/mL).^4,5^ While the preoperative daily pill burden in our study was comparable to others,^1,6^ this is the first study to quantify the postoperative pill burden. Although the excessive postoperative pill burden decreases over time, there is still a modest increase at 6 months.

Our study is limited by (1) a substantially higher preoperative PTH level in our patient population than previously recommended biochemical thresholds, which may impact the severity of postoperative hypocalcemia, and (2) inconsistent medication reconciliation documentation after discharge. Higher preoperative PTH values may be associated with greater challenges with calcium homeostasis postoperatively and warrant additional study.

In conclusion, this study highlights the substantial postoperative pill burden after parathyroidectomy for SHPT. To alleviate some of the excessive pill burden, providers should consider dose consolidation. This new knowledge on pill burden should enhance shared decision-making conversations between patients and clinicians to help set postoperative expectations.
